# Through the eyes of grandparents: an in-depth exploration of the nexus between grandchild caring and the psychological well-being of older grandparents

**DOI:** 10.1186/s12877-024-04998-z

**Published:** 2024-05-05

**Authors:** Papai Barman, Harihar Sahoo

**Affiliations:** 1 Department of Family & Generations, International Institute for Population Sciences, Mumbai, 400088 India; 2 International Institute for Population Sciences Department of Development Studies, Mumbai, India

**Keywords:** Grandchild caring, Psychological well-being, Skipped generation Household (SGH), Multi-generation Household (MGH), Indepth Interview, Thematic analysis, Content analysis, Caring Intensity, India

## Abstract

**Background:**

Grandchild caring has positive as well as negative impact on the grandparents’ psychological well-being and the findings are varied by culture and country.

**Methods:**

Present study was intended to understand the relationship between caring for grandchildren and psychological well-being of grandparents living in skipped (SGH) and multi-generational households (MGH) in Indian demographical context. The present research involved In-depth Interviews (IDI) focusing on grandparents above 60 and grandchildren below 18, where the elder played a crucial role in caregiving. The study area was Malda, a district of West Bengal in India. Purposively 24 IDIs were selected. Psychological well-being was measured using open-ended questions. Thematic and content analyses were adopted to understand the perspective of grandparents.

**Results:**

Most of the grandparents from SGH reported depression word frequently, while grandparents from MGH reported happy. In the content analysis, grandparents from SGH expressed tension, mental turmoil, and worry about grandchild’s future. On the contrary, grandparents from MGH expressed happy, companionship, and worry about grandchild’s future. Further, full time caring, compulsive reason behind grandchild caring, and working status were linked with living in SGH and grandchild caring, which were in turn connected with deteriorate psychological health. However, in MGH, a different scenario was observed, most grandparents were partially and non-compulsively engaged in grandchild caring and had expressed positive mental health.

**Conclusions:**

The Findings provide an intervention implication, particularly in the context of India’s ageing population and their well-being by acknowledging the influence of household structure, caring intensity, motive behind grandchild caring, and working status on their psychological health. Understanding the importance of these key factors may help the policy maker and the individual to incorporate the most effective intervention to achieve sustainable development goal 3 and healthy ageing.

**Supplementary Information:**

The online version contains supplementary material available at 10.1186/s12877-024-04998-z.

## Introduction

Grandparents’ psychological health has received much research attention, particularly in light of their later-life caring responsibilities. In addition to characteristics like depression, loneliness, happiness, and life satisfaction, psychological well-being also has an impact on overall health [1]. The selection of a person’s later-life living arrangements has grown to be a substantial subject of research interest in healthy ageing and psychological well-being. Regarding living arrangements, skipped-generation households (SGH) and multi-generational households (MGH) are critical areas of research. SGH involves grandparents and grandchildren living together without the child’s parents [2–4]. MGH, however, includes three generations and often more family members. SGH caregiving where family crisis, unplanned parenthood, and love are present altogether often entails surrogate or custodial parenting, while MGH caregiving where parental love and the caregiving roles assumed by grandparents is mostly rooted love and affection leans towards co-parenting and non-full-time caregiving [5].

Further, life-living decisions are influenced by various social, physical, financial, and familial considerations. Older individuals frequently prefer MGH living for a relaxed lifestyle, but obstacles like social or financial crises often force them into SGH living. In caring for grandchildren in living SGH, one may expect a significant impact on grandparents’ mental health. Numerous worldwide studies have reported adverse effects on grandparents’ psychological well-being in caregiving roles (2, 3, 6–7). Conversely, some studies have found positive outcomes associated with grandchild caregiving [3, 6–9]. Notably, the effect tends to be positive in Asian and African contexts, emphasizing strong familism, while Western studies mostly report a negative impact [6, 10–12]. Considering cultural and contextual factors, these divergent findings highlight the need for further research to clarify the relationship between grandchild caregiving and psychological well-being.

Additionally, in a nation like India, where familism and collectivism are strongly held beliefs, the dynamics of household structure, particularly in SGH, may have a different impact [13]. Furthermore, given that co-parenting is frequently linked with MGH, whereas intensive or full-time caregiving is frequently linked with SGH, one might infer the significance of caring hours and variations in their impact on grandparents in SGH and MGH. The “second shift” phenomenon, in which grandparents work and provide care, may also add to the burdens already placed on those living in senior housing [14–18].

Previous studies discussed various reasons for grandchild caregiving, but few examined its significance in determining grandparents’ mental health [4, 17]. Understanding mental health among grandparents requires their active participation in grandchild caregiving. While some grandparents do this responsibility out of pure love, others find themselves compelled by necessity. As a result the impact on the grandchildren’s well-being differs depending on the diverse motivations and circumstances. For example, Xu et al. (2017), reported that majority of grandparents are involved in the grandparenting for intergenerational support, bond, and love [12]. Chen & Liu (2012) also mentioned that majority of grandparents from China are involved in grandchild caring due to alleviate the burden of adult members [18]. Studies also mentioned about the involvement in grandchild caring and psychological health [5, 19]. The situation is frequently enjoyable for grandparents who voluntarily look after their grandchildren. On the other hand, providing unintentional care might be difficult, particularly in SGH without assistance [20]. Many Asian nations anticipate grandparents to co-parent and live in MGHs, but unexpected family crises might induce a sense of obligation. The impact of grandparent caregiving on psychological health must be understood to appreciate its causes and motivations fully.

In India, where the ageing population is expanding quickly and SGH and MGH are common, it is essential to comprehend the relationship between caring for grandchildren and psychological well-being. Further, few studies have examined grandparents’ perspectives on caring for their grandchildren and their mental health. To fill in research gaps, a ground-breaking study conducted in India looked at grandparents’ views, levels of caregiving, motives, and work status. The findings, the first of their sort worldwide, provide information that can improve our comprehension when considering regional and cultural differences. By considering elements like caring intensity, motives, and work status in later life, this knowledge can help enhance the well-being of elderly grandparents.

## Data and methodology

### Data

Our primary research involved In-depth Interviews (IDI) focusing on grandparents aged 60 and above and grandchildren below 18, where the elder played a crucial role in caregiving. IDI is a qualitative research technique involving intensive individual interviews with a few respondents to explore their perspectives on a particular idea, problem, or situation. IDIs were conducted to collect more detailed information on perceptions and experiences. The study area was Malda, a district of West Bengal in India. The current concern revolves around the specificity of the Malda district. This is due to the underlying assumption in the present study that the expectations, the impact of grandchild care, and the overall experience of older grandparents from any region in India will remain consistent, irrespective of whether grandparenting occurs in the western, eastern, northern, or southern parts of the country.

### The participants and procedures

Participants for the in-depth interview were selected purposively; for this purpose, we first started to identify the potential sources of the respondents by talking to the village resource person, where I went through an initial contact and introduction. Then, pre-selection screening was done using the following selection criteria which were developed based on the study objectives. The inclusion criteria were as follows (a) participants must be aged 60 and above, (b) participants must have at least one grandchild aged below 18 years, (c) participants must be responsible for grandchild caring, and ca. for SGH: adult member must not present in the household at least 6 months. The exclusion criteria were as follows: (a) participants should not suffer from chronic disease or illness, (b) participants should not be bedridden, and (c) participants should be household members and have resided in the household for the last six months. To ensure unbiased selection, a deliberate effort was made to avoid known or familiar households or individuals, thereby minimizing potential biases in participant selection. Then, Informed consent was presented in front of each participant, clearly outlining the research purpose, procedures, potential risks, benefits, and their rights. Then we assured confidentiality and the voluntary nature of participation (prefer supplement file for more details). After getting consent, we collaboratively decide on suitable dates, times, and locations for the in-depth interviews. Open-ended questions were used to elicit rich and detailed responses from participants. These questions were designed to explore various dimensions, including reasons for grandchild care, motivating factors, household dynamics, financial considerations, social engagements, and mobility limitations. Ensuring diversity in participant characteristics and experiences an ample place was explored. A notebook and a Tape recorder were used to record the information during the in-depth interview. We stopped collecting information when we found similar information, meaning that information was reached at saturation point—where new interviews cease to yield substantially further information—indicating that data collection is complete.

### Sample size

The final study sample was 24 IDIs. Of these, 12 IDIs were from skipped generation, and another 13 were from multi-generation households.

### Psychological well-being

Psychological well-being was measured using open-ended questions that were asked to the grandparents “*How do you feel about caring, sometimes it is a lot of responsibility, burden and at the same time it is a pleasure to spend time and get social, physical, and mental support with grandchild? What is in your case?*” We tried to comprehend the responses and experiences about grandchild caring about psychological health.

### Data analysis

We utilised IDI information as qualitative data analysis through thematic and content analyses, providing a comprehensive understanding of the grandparents’ perspective. An open-ended question was utilised. Audio recordings were transcribed for the thematic and content analyses [21, 22]. For thematic analysis, we drew a word cloud using Nivo 12, and for the content analysis, we read the interview transcripts multiple times to become familiar with the content. It helps to understand better the transcripts and sense of the participants’ experience. Numerous times reading, we tried to make codes of emerging ideas or issues. Further, each of the codes was compared and some categories were created based on the similarity among the codes, then, we tried to comprehend the grandparents’ perspective from the analysis. These procedures were separately done for SGH and MGH.

## Result

Table 1 presents the sample characteristics. Among the 24 participants, 45.8% [11] were from SGH and 154.2% [13] were from MGH. Most respondents were female and from Hindu, Other social category, and rural areas. It also revealed that a higher proportion of individuals in MGH were currently married, accounting for 61.5% [8], whereas more widowed individuals were found in SGH, representing 54.5% [6]. Regarding religion, there was an even distribution between Hindu and Muslim populations in SGH, while in MGH, Hindus constituted 55.6% [10] of the sample. Social category showed significant variation, with Scheduled Tribes (ST) exclusively present in SGH at 100% [4], and Scheduled Castes (SC) being more common in MGH, making up 77.8% [7] of that group. In terms of place of residence, rural areas were more prevalent in SGH at 52.4% [11], while urban areas were exclusively associated with MGH at 100% [3]. Further, the mean age of the participants was 69 for SGH and 65 for MGH, reflecting younger older. The mean number of grandchildren for both households was 2.2 for MGH and 2.4 for SGH. Additionally, the mean age of the youngest grandchild among grandparents was 5.4 for MGH and 5.3 for SGH, reflecting no significance differences in the number of grandchildren and age of the youngest grandchild between SGH and MGH.

**Table 1:** Sample characteristics of IDI participants

Background	SGH %(*n*)	MGH %(*n*)	Total
Sex			
Male	66.7 [2]	33.3 [1]	3
Female	42.9 [9]	57.1 [12]	21
Marital Status			
Currently married	38.5 [5]	61.5 [8]	13
Widowed	54.5 [6]	45.5 [5]	11
Religion			
Hindu	44.4 [8]	55.6 [10]	18
Muslim	50.0 [3]	50.0 [3]	6
Social category			
SC	22.2 [2]	77.8 [7]	9
ST	100.0 [4]	0.00 (0)	4
Other	45.5 [5]	54.5 [6]	11
Place of residence			
Rural	52.4 [11]	47.6 [10]	21
Urban	0.00 (0)	100.0 [3]	3
Total	45.8 [11]	54.2 [13]	24
Age of grandparent (mean)	65.5	66.5	
Number of grandchildren (mean)	2.4	2.2	
Age of youngest grandchild (mean)	5.3	5.4	

Source: computed using IDI information

**Fig. 1:**
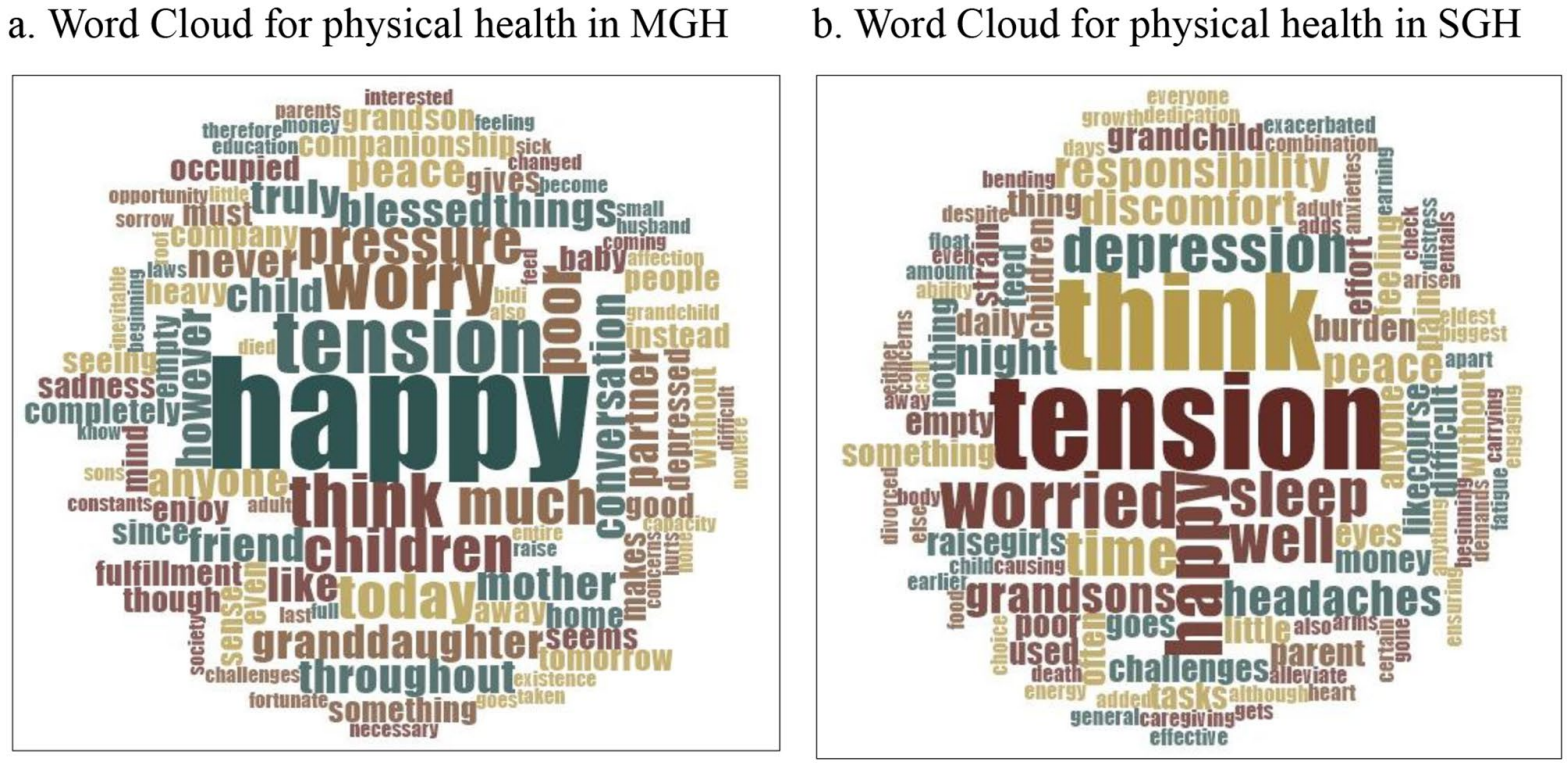
Word Cloud for time management when grandparent is responsible for GCC in MGH and SGH Source: computed using IDI information

**Fig. 2:**
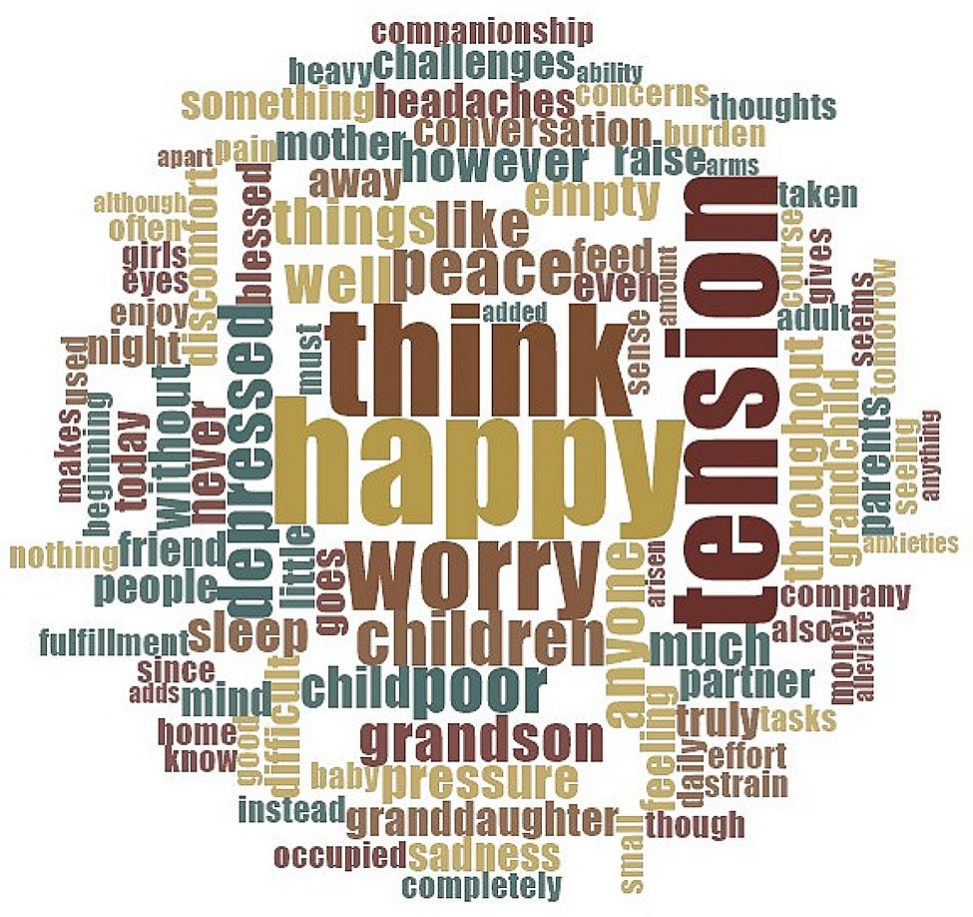
Word Cloud for time management in both the MGH and SGH Source: computed using IDI information

Figure 1 shows a word cloud of the key terms related to the mental health of grandparents staying in MGH and SGH. The five most frequent terms in MGH, from IDIs, were happy, tension, think, worry, and children (Fig. 1.a). In contrast, the five most frequent terms were tension, think, worried, happy, and depression in SGH (Fig. 1.b). A significant difference was observed in the five most frequent terms among the grandparents staying in both MGH and SGH. Happy, tension, think, worry, and children were the top five terms across all IDIs (Fig. 2).

### Grandparents’ perspective in SGH

**Fig. 3:**
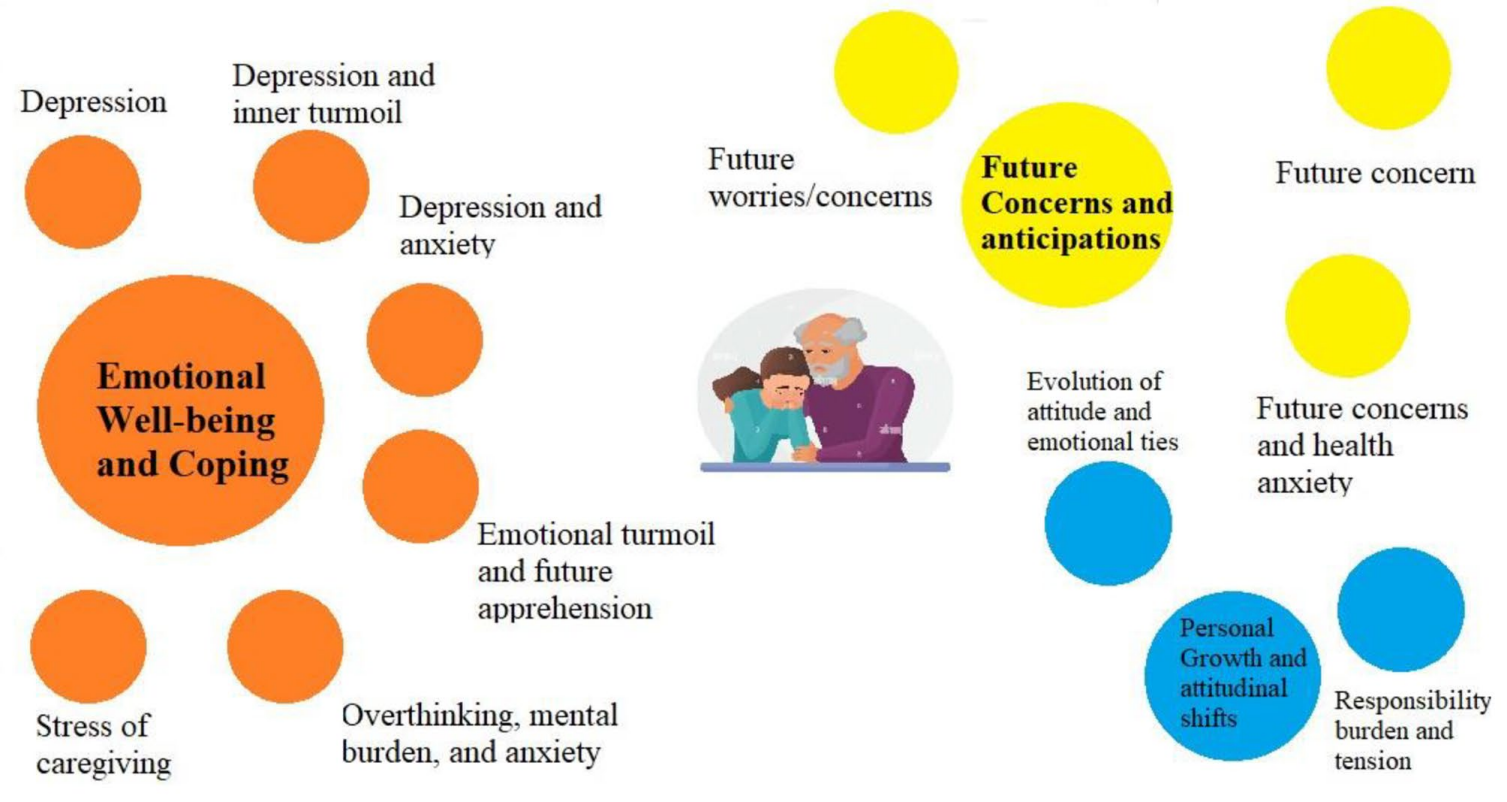
Visualizing qualitative data from Open-ended questions Source: computed using IDI information Note: Circles are proportional to the number of responses of each category and issue

We also made an effort to understand the responses from IDIs residing in SGH and MGH utilising content analysis. Firstly, considering the responses from IDIs living in SGH, the qualitative data collected from the open-ended question “How do you feel about caring, sometimes it is a lot of responsibility, burden and at the same time it is a pleasure to spend time and get social, physical, and mental support with grandchild? What is in your case?” were examined and transformed into categories and issues presented in Table 2; Fig. 3. The information was coded and categorised to identify common patterns. The classification produced by the analysis is as follows:

### Emotional health and coping

This category focuses on the emotional experiences and coping mechanisms of the participants. It includes issues related to depression, anxiety, emotional turmoil, and the stress of caregiving. Participants described feelings of depression and anxiety, and the emotional toll caregiving can take on them. They also reflected on the stress they experienced while caregiving and how they did cope with these emotional challenges.

I had no choice…feel depression while thinking about them.

In my case, taking care of my grandchild has…physical problems and depression.

*“I’m in trouble, feeling depressed and problem.“*

It entails a significant amount of responsibility… despite my efforts to stop worrying, I find it challenging to do so.

### Future concerns and expectation

This category revolves around participants’ concerns and anticipations regarding the future of their caregiving role. Issues in this category include worries about the grandchildren’s future, health anxiety related to the future, and general concerns about what lies ahead. Participants expressed tensions and uncertainties about how their caregiving responsibilities will evolve over time.

I always have a tension about what will happen to the grandchildren and what will be their future; their life is over.

*“I have tension that what will be in future with her, who will take care of her.“*

*“After the death of the eldest son.,I was worried about their future and nothing else.”*

### Personal growth and attitudinal shifts

This category explores personal growth and changes in participants’ attitudes as a result of their caregiving experiences. The evolution of attitudes and emotional ties is highlighted, where participants discussed how their feelings and perceptions have changed over time. Additionally, there was an exploration of the burden of responsibility and tension that comes with caregiving, particularly when there is a lack of external support.

*“I don’t feel lonely because I’m used to it. Earlier I used to have problem in the beginning of the started caring, I didn’t like it, I wanted peace. Now I don’t think it’s a problem, if the girls aren’t here, I look for them. I like to look for them, feed them, and always see in front of my eyes. Without the girls, the I feel empty house.*

*“Caring a kid without help of adult child and engaging in earning process, I feel lot of responsibility and tension. Grandchild caring at this age I find it as burden.”*

**Table 2:** Categories and issues from the open-ended question “How do you feel about caring, sometimes it is a lot of responsibility, burden and at the same time it is a pleasure to spend time and get social, physical, and mental support with grandchild? What is in your case?” in SGH

Category	Code (Issues)	Example
Emotional health and coping	Depression and anxiety	“I had no choice…feel depression while thinking about them.”
	Depression	“In my case, taking care of my grandchild has…physical problems and depression.”
	Depression and inner turmoil	“I’m in trouble, feeling depressed and problem.”
	Emotional turmoil and future apprehension	“Happiness and sadness are always there, does anyone stay happy throughout their life? There will be problem and worry.”
	Overthinking and mental burden, anxiety	“It entails a significant amount of responsibility… Despite my efforts to stop worrying, I find it challenging to do so.”
	Stress of caregiving	“Of course, I’m happy to have the kids in front of me, but at the end of the day raising three small kids are really very difficult for me… this leads me to have stress.”
Future concerns and expectations	Future worries/concerns	“I always have a tension about what will happen to the grandchildren and what will be their future; their life is over.”
	Future concerns and health anxiety	“I have tension that what will be in future with her, who will take care of her.”
	Future concern	“After the death of the eldest son.,I was worried about their future and nothing else.”
Personal growth and attitudinal shifts	Evolution of Attitude and Emotional Ties	“I don’t feel lonely because I’m used to it. Earlier I used to have problem in the beginning of the started caring, I didn’t like it, I wanted peace. Now I don’t think it’s a problem, if the girls aren’t here, I look for them. I like to look for them, feed them, and always see in front of my eyes. Without the girls, the I feel empty house.
	Responsibility burden and tension	“Caring a kid without help of adult child and engaging in earning process, I feel lot of responsibility and tension. Grandchild caring at this age I find it as burden.”

Source: computed using IDI information

### Grandparents’ perspective in MGH

**Fig. 4:**
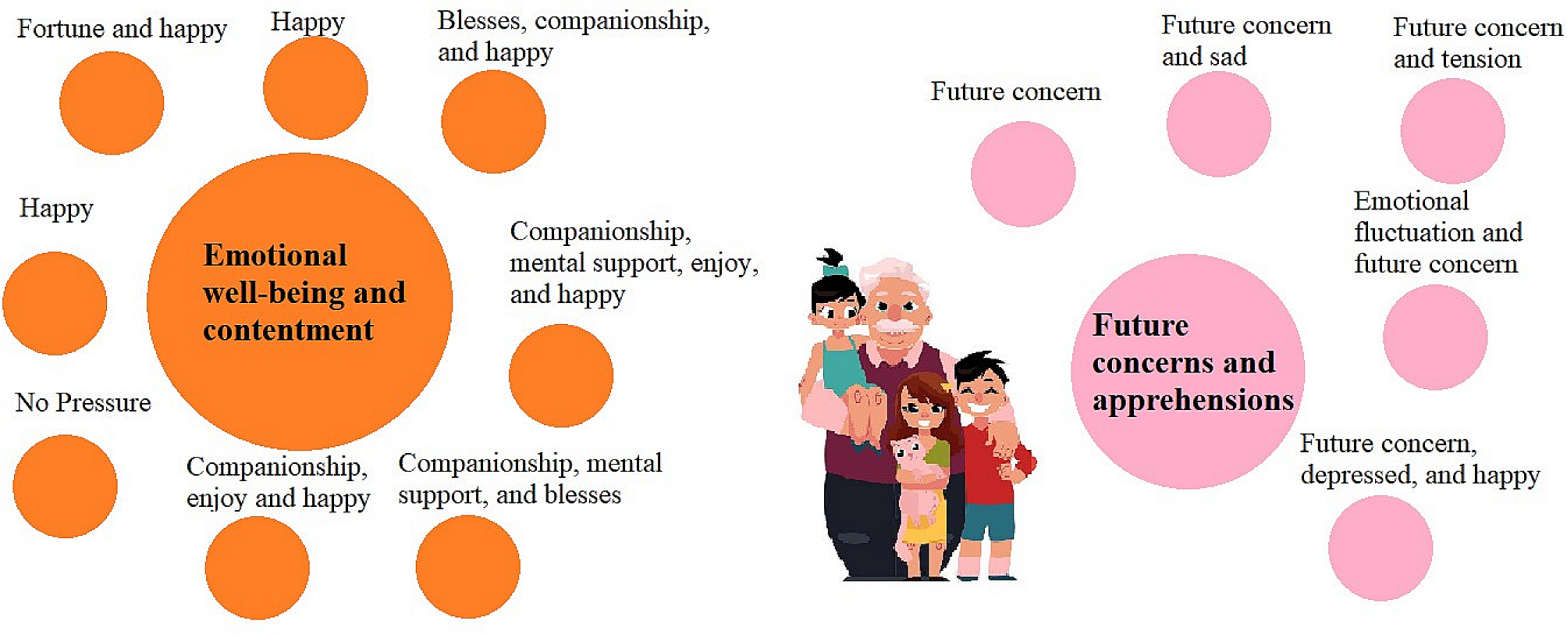
Visualizing qualitative data from Open-ended questions Source: computed using IDI information Note: Circles are proportional to the number of responses of each category and issue

Considering the responses from IDIs living in MGH based on the the open-ended question “How do you feel about caring, sometimes it is a lot of responsibility, burden and at the same time it is a pleasure to spend time and get social, physical, and mental support with grandchild? What is in your case?” was analyzed and organized into categories and issues in Table 3; Fig. 4. The data was coded to identify common themes and patterns. The analysis resulted in the following categorization:

### Emotional health and contentment

This category encompasses responses that reflect positive emotions, contentment, and a sense of well-being experienced by the respondents while caring for their grandchildren. Caregivers in this category express feelings of happiness, companionship, and fulfillment in their role.

*“I feel fortunate to get an opportunity for grandchild caring. I am happy with my grandson.“*

*“I don’t have any tension with the children, but I worry when they are sick. I feel happy it when grandchildren are good.“*

*“I feel like blessed after getting a friend and a conversation partner.“*

*“I don’t feel any pressure or a heavy responsibility; instead, I truly enjoy taking care of and spending time with my grandchildren…I am happy with them.”*

### Future concerns and apprehensions

This category reflects the concerns and apprehensions that respondents have about the future, especially when it comes to the well-being and care of their grandchildren in their absence. Caregivers in this category worry about what will happen to their grandchildren when they are no longer able to provide care and express a mix of emotions, including sadness, tension, and even depression when thinking about the future.

*“A big tension is that if something happens to me today, what will happen to these grandchildren? Their mother has not interested or affection on them.“*

*“I worry that the child’s father has died, and after having a mother, there is no mother, what will happen in the future, I worry about these things.“*

*“I keep thinking all the time about how these grandchildren will grow and spend their life without a father. We are alive today, what will happen to the grandchildren tomorrow when we die, who will take care of my daughter and grandchildren…always tension in my mind.”*

**Table 3:** Categories and issues from the open-ended question “How do you feel about caring, sometimes it is a lot of responsibility, burden and at the same time it is a pleasure to spend time and get social, physical, and mental support with grandchild? What is in your case?” in MGH

Category	Codes Issues)	Example
Emotional health and contentment	Fortune and happy	“I feel fortunate to get an opportunity for grandchild caring. I am happy with my grandson.”
	Happy	“I don’t have any tension with the children, but I worry when they are sick. I feel happy it when grandchildren are good.”
	Blesses, companionship, and happy	“I feel like blessed after getting a friend and a conversation partner.”
	Companionship, mental support, enjoy, and happy	“I don’t feel any pressure or a heavy responsibility; instead, I truly enjoy taking care of and spending time with my grandchildren…I am happy with them.”
	Companionship, mental support, and blesses	“Even though we have son and daughter-in-law, they don’t get time to stay at home, the house seems completely empty. However, I feel like blessed after getting a friend and a conversation partner.”
	Companionship, enjoy and happy	“I truly enjoy taking care of and spending time with my grandchildren. Since my husband and children are occupied with their work, and I don’t have much work to do, spending time with the grandchildren and taking on this responsibility gives me companionship and a sense of fulfillment. I am happy with them.”
	Happy	“I don’t feel any pressure, physical problem or tension. Now, I’m doing well with these grandchildren.”
	No Pressure	“I don’t feel any pressure”
Future concerns and apprehensions	Future concern	“A big tension is that if something happens to me today, what will happen to these grandchildren? Their mother has not interested or affection on them.”
	Future concern and sad	“I worry that the child’s father has died, and after having a mother, there is no mother, what will happen in the future, I worry about these things.”
	Future concern and tension	“I keep thinking all the time about how these grandchildren will grow and spend their life without a father. We are alive today, what will happen to the grandchildren tomorrow when we die, who will take care of my daughter and grandchildren…always tension in my mind.”
	Emotional fluctuation and future concern	“In the beginning I was happy to raise my granddaughter. Now it is changed into a tension when I think about my granddaughter’s future without parents. We become old and don’t have so much capacity to give her a good life and education.”
	Future concern, depressed, and happy	“I feel happy now because it is the last company. There is none who will give such company. I feel sometimes depressed by thinking that what will happen when we will not be there. I feel depressed but looking at the face of the granddaughter, nowhere to go. I always stay in tension about her future.”

Source: computed using IDI information

### Caring hours and mental health

The study revealed an association between the amount of time dedicated to caring for grandchildren and the mental well-being of grandparents. In SGH, a substantial number of grandparents discussed the hours they devoted to grandchild care and their emotional experiences at the end of each day. Extensive caregiving hours were consistently linked to heightened tension, anxiety, and mental distress. Grandparents in this group often found themselves preoccupied with concerns about their grandchildren’s future, leading to symptoms of depression and persistent trouble. Physical manifestations of this mental burden included frequent headaches and sleep difficulties. In contrast, in MGH, where caregiving hours were limited, grandparents reported overall happiness and contentment. The reduced intensity of caregiving led to a more positive mental state.

### Reasons behind grandchild caring and mental health

The reasons driving grandparent caregiving differed significantly between SGH and MGH, with corresponding effects on their mental well-being. In SGH, where caregiving stemmed from compulsive situations like an adult child death or a son-in-law’s refusal to care, grandparents commonly reported experiencing heightened tension, depression, and mental distress. The profound concern for their grandchildren’s future weighed heavily on their minds, resulting in prolonged mental burdens and accompanying physical symptoms such as headaches and sleep disturbances. Conversely, in MGH, where caregiving was motivated by a desire to assist adult children or out of love for caregiving, grandparents expressed feelings of happiness and contentment, with no reported mental distress.

### Work and mental health

The study further highlighted the influence of grandparents’ roles and responsibilities on their mental well-being, particularly in SGH and MGH. In SGH, where grandparents balanced caregiving with earning and household chores, a common theme emerged – the experience of tension, anxiety, and persistent mental distress. The multifaceted nature of these responsibilities compounded to create lasting mental burdens, ultimately contributing to depression and the emergence of physical symptoms like headaches and sleep disturbances. In contrast, within MGH, where grandparents primarily focused on household chores without the additional burden of earning, they reported experiencing happiness and contentment. They viewed caregiving as a fortunate opportunity, fostering positive mental health without accompanying distress.

## Discussion

The present study using the In-depth-interview information was intended to understand the association between grandchild caring at later life and psychological health. It was understood that cross-sectional studies are limited by their inability to explain the causation and temporality and the non-random nature of the selection bias; therefore, we just fixed the study on looking association. Findings indicated that most grandparents living in MGH expressed happiness, whereas in SGH, the majority expressed tension, reflecting the differences in the perspective of grandchild caring in later life, especially in SGH and MGH.

Further, we tried to understand the grandparents’ perspective in a better way, using content analysis separately for SGH and MGH. Findings indicated that grandparents living in SGH had no choice, often felt depression and mental trouble. Most of the grandparents expressed depression, inner turmoil, overthinking, anxiety, and stress under the “emotional health and coping” section. Similarly, under the section “future concerns and expectations,” most reported worry about future concern. On the contrary, grandparents living in MGH expressed fortune, happy, blessed, mental support, and no pressure under the section of emotional health and contentment”. Majority also reported future concern of grandchild and tension under the section “future concerns and apprehensions”. Within SGH, grandparents often shoulder the caregiving burden without the presence of their children, a factor that may lead to increased stress and emotional strain. The absence of adult children in SGH places grandparents in a surrogate parenting role, necessitating adjustments that may adversely affect their psychological health. These findings are consistent with previous literature suggesting that caregiving without the support of one’s adult children, especially in such type of household, may lead to heightened stress levels, exacerbating feelings of depression and loneliness [23, 24]. The findings are also consistence with previous literatures reported that older people living in joint household are having good mental health than those living alone or nuclear family [25, 26]. A study based on India mentioned that around 9% of older people living in depression and the percentage tends to increase around 13% among those living alone and decrease 8% among those residing in someone, reflecting the importance of the other family members [27].

We also tried to understand the linkage between caring hour or intensity and the psychological health of the grandparents. Findings revealed a linkage between caring hour or intensity and psychological health of the grandparents, most of the grandparents from SGH expressed full-time caring responsibility heightened levels of tension, anxiety, and mental distress. On the contrary, most of grandparents from MGH expressed part time responsibility, happiness, and contentment. Full-time caring deteriorates mental health and partial responsibility increases the mental health.

Previous studies also mentioned that custodial or full-time caring has a negative effect on grandparent’s mental health (23–24). These findings highlighted the significance of accounting for caring hour or intensity when evaluating its impact on grandparents’ mental health. The finding reflects the acceptance of the role-strain theory, which suggests multiple roles and distress. Moreover, living in SGH without the help of other members, grandchild caring and household management responsibility, grandparents and parental roles sometimes creates mental pressure and burden and in turn suffer from poor psychological health [30–33]. Aligning with the previous studies, the finding of the current research underscored the importance of the type of caring hour responsibility.

Findings also highlighted the significant linkage between the reason behind grandchild caring and psychological health. Grandparents responsible for grandchild caring due to compulsive situations (for example, “had no choice”) experienced heightened tension, depression, and mental distress. This emotional strain may contribute to increased poor mental health. A study by Goodman and Silverstein, (2006) also mentioned that due to grandchild caring and over-strain, grandparents often feel depression [28, 29]. It may also be that due to compulsive situations there is a lack of choice and autonomy, which in turn may lead to feelings of frustration, which are associated with higher rates of depression and life dissatisfaction [35].

Conversely, in MGH, a significant portion of grandparents caring for their grandchild due to help and love expressed happiness without mental distress, reflecting the importance of the reason behind grandchild caring to understand the relationship between grandchild caring and psychological health. Therefore, understanding caregiving motives is crucial, especially in SGH, where challenging circumstances may require additional mental health support. It also may be that compulsive caregiving may limit grandparents’ opportunities for social engagement and leisure activities, leading to social isolation and depression. Moreover, poor mental health is often linked with a lack of social interaction and support, contributing to adverse mental health outcomes [36]. One possible reason may also be that compulsive caregiving and family crises are often related to financial burdens, especially when grandparents are not financially prepared for this role. Financial stress is a well-known contributor to depression and life dissatisfaction [32–34].

Furthermore, findings revealed an association between working status and psychological health, reflecting that grandparents who were currently engaged in working were experiencing more deteriorate psychological health than those who did not. In SGH, most grandparents engaged in caregiving with earning and household chores and the majority of them reported experience of tension, anxiety, and persistent mental distress. In contrast, in MGH, most grandparents primarily focused on household chores without the additional burden of earning, they reported experiencing happiness and contentment. They viewed caregiving as a fortunate opportunity, fostering positive mental health without accompanying distress. In SGH, juggling multiple responsibilities appears to be associated with adverse mental outcomes, highlighting the need for support and resources to alleviate these challenges. This observation draws attention to the ‘second shift,’ whereby grandparents in SGH must juggle caregiving responsibilities and employment. The ‘second shift’ phenomenon poses a considerable challenge to older grandparents, as they are required to fulfill their caregiving duties while simultaneously managing employment obligations caregiving [14–18, 37–39, 37–39]. This duality may increase stress and reduce opportunities for self-care and social engagement. Consequently, the psychological well-being of these individuals may be compromised. However, findings underscored the critical role of grandparents’ working status on the relationship between grandchild caregiving in various households and psychological health.

The present study, like other studies, had some limitations: (1) Due to a lack of previous health status information for older individuals, the study was unable to control the effects of past health conditions. (2) The study relied on cross-sectional primary information. Longitudinal information would have provided more accurate insights into the grandparent’s well-being. (3) The study only focused on the grandparent who was prime to take the responsibility out of two or more grandparents in the same households.

### Policy and intervention implications

The study’s findings carry substantial importance in healthy ageing because the analyses were based on the perspective of grandparents using open-ended questions where grandparents were open to expressing their feelings rather than just using closed-ended questions. The Findings provide an intervention implication, particularly in the context of India’s ageing population and the well-being of older grandparents by acknowledging the influence of household structure, caring intensity, motive behind grandchild caring, and working status on their psychological health. Culturally sensitive programs that respect local norms and values can be instrumental in improving the psychological health of older grandparents. Understanding the importance of these key factors may help the policy maker and the individual to incorporate the most effective intervention to achieve sustainable development goal 3 and healthy ageing.

## Conclusion

In conclusion, this study provides insightful intuitions into the nuanced interaction between raising grandchildren and elder grandparents’ psychological health. The findings highlight the importance of household structure, caring intensity, motivation, and working status in determining the psychological health of older grandparents. It is imperative to acknowledge these essential factors to adopt effective policies and interventions aiming at improving psychological well-being on the line of healthy ageing in India and similar contexts globally.

### Electronic supplementary material

Below is the link to the electronic supplementary material.

Supplementary Material 1

## Data Availability

The datasets generated and/or analysed during the current study are not publicly available due to the necessity of safeguarding the participants’ confidentiality and privacy throughout the study but are available from the corresponding author on reasonable request.

## References

[1] Chang Y, Huang J (2020). Impacts of intergenerational care for grandchildren and intergenerational support on the psychological well-being of the elderly in China. Rev Argentina Clin Psicol.

[2] Hughes ME, Waite LJ, LaPierre TA, Luo Y (2007). All in the family: the impact of caring for grandchildren on grandparents’ health. Journals Gerontol Ser B Psychol Sci Soc Sci.

[3] Williams L. Health of Grandmothers Raising Children of the Crack Cocaine Epidemic Author (s): Kathleen M. Roe, Meredith Minkler, Frances Saunders and Gregg E. Thomson Published by : Lippincott Williams & Wilkins Stable URL : https://www.jstor.org/stable/3766562. 1996;34(11):1072–84. 10.1097/00005650-199611000-00002 8911424

[4] Mutchler J, Baker LA. Grandparent Care in the Asian Population Grandparent Care in the Asian Population. 2002.

[5] Goodman C, Silverstein M (2002). Grandmothers raising grandchildren: family structure and well-being in culturally diverse families. Gerontologist.

[6] Silverstein M. and DZ. Grandparents caring for grandchildren in rural China: consequences for emotional and cognitive health in later life Grandparents caring for grandchildren in rural China : consequences for emotional and cognitive health in later life. Aging Ment Health [Internet]. 2021;25(11):2042–52. 10.1080/13607863.2020.1852175 10.1080/13607863.2020.1852175 33251822

[7] Silverstein M, Cong Z, Li S. Intergenerational transfers and living arrangements of older people in Rural China: consequences for Psychological Well-Being. 2006;61(5):256–66. 10.1093/geronb/61.5.s256 16960239

[8] Kim J, Park E, Choi Y, Lee H, Lee SG (2017). The impact of intensive grandchild care on depressive symptoms among older koreans. Int J Geriatr Psychiatry.

[9] Arpino B, Bordone V (2014). Does grandparenting pay off? The effect of child care on grandparents’ cognitive functioning. J Marriage Fam.

[10] Blustein J, Chan S, Guanais FC (2004). Elevated depressive symptoms among caregiving grandparents. Health Serv Res.

[11] Grundy EM, Albala C, Allen E, Dangour AD, Elbourne D, Uauy R (2012). Grandparenting and psychosocial health among older chileans: a longitudinal analysis. Aging Ment Heal.

[12] Xu L, Tang F, Li LW, Dong XQ. Grandparent Caregiving and Psychological Well-being among Chinese American older adults — the roles of Caregiving Burden and pressure. 2017;72:56–62. 10.1093/gerona/glw186 28575256

[13] Silverstein M, Marenco A. How Americans enact the grandparent role across the family life. 2001;6052.

[14] Hochschild AR, Machung A. The Second Shift (Excerpts). 2003;9780143120.

[15] Hochschild A. The second shift: Working parents and the revolution at home. 1989.

[16] Anderson SG, Liu M, Liao M (2013). Subsidized child care by grandparents: profiles of caregivers in an emerging Public Service Context. J Women Aging.

[17] Choi SW, Zhang Z (2018). Grandparenting and Self-Rated Health among older Korean Women. Res Aging.

[18] Chen F, Liu G (2012). The health implications of grandparents caring for grandchildren in China. Journals Gerontol - Ser B Psychol Sci Soc Sci.

[19] Fuller-Thomson E, Minkler M (2001). American grandparents providing extensive child care to their grandchildren: prevalence and profile. Gerontologist.

[20] Wen M, Ren Q, Korinek K, Trinh HN. Living in skipped generation households and happiness among middle-aged and older grandparents in China. Soc Sci Res [Internet]. 2019;80(January):145–55. 10.1016/j.ssresearch.2019.01.004 10.1016/j.ssresearch.2019.01.004 30955552

[21] Alves M, Siqueira M, Gonçalves JP, Mendonça VS, Kobayasi R, Arantes-costa FM et al. Relationship between metacognitive awareness and motivation to learn in medical students. 2020;1–10. 10.1186/s12909-020-02318-8 PMC7602298 33126882

[22] Tempski P, Bellodi PL, Paro HBMS, Enns SC, Martins MA, Schraiber LB. What do medical students think about their quality of life ? A qualitative study. 2012. 10.1186/1472-6920-12-106 PMC3527341 23126332

[23] Baker LA, Silverstein M (2008). Preventive health behaviors among grandmothers raising grandchildren. Journals Gerontol - Ser B Psychol Sci Soc Sci.

[24] Leder S, Grinstead NL, Torres E (2007). Grandparents raising grandchildren: stressors, social support, and health outcomes. J Fam Nurs.

[25] Patil A (2022). Neuroticism amongst elderly staying in joint families and nuclear families. Indian J Ment Heal.

[26] Akbar S, Tiwari SC, Tripathi RK, Pandey NM, Kumar A (2018). Prevalence of psychiatric illness among residents of old age homes in Northern India. J Neurosci Rural Pract.

[27] Srivastava S, Debnath P, Shri N, Muhammad T. The association of widowhood and living alone with depression among older adults in India. Sci Rep [Internet]. 2021;11(1):1–13. 10.1038/s41598-021-01238-x 10.1038/s41598-021-01238-x PMC8568934 34737402

[28] Li H, Gan L, Xu D (2022). Long-term impact of Grandchild Caregiving trajectories on Depression in Middle-aged and older Chinese people: a longitudinal study. Int J Aging Hum Dev.

[29] Hong Y, Xu W. Continuity and changes in grandchild care and the risk of depression for Chinese grandparents: new evidence from CHARLS. Front Public Heal. 2023;11(August). 10.3389/fpubh.2023.1217998 PMC10435994 37601176

[30] Dong X, Ling H, Yang T, Wang K (2023). Grandchild care and life satisfaction of older adults: empirical evidence from China. Front Psychol.

[31] Goode WJ (1960). A theory of role strain. Am Sociol Rev.

[32] Pearlin LI, The Sociological Study of Stress Author (s). Sep.,. : Leonard I. Pearlin Source : Journal of Health and Social Behavior, 1989, Vol. 30, No. 3 (Sep., 1989), pp. Published by : American Sociological Association Stable URL : https://www.jstor.org/s. 1989;30(3):241–56. 2674272

[33] Sieber SD (1974). Toward a theory of role accumulation. Am Sociol Rev.

[34] Goodman CC. Ethnic and Racial Differences in and Coparenting Families. 2006;1605–26.

[35] Minkler M, Fuller-Thomson E (1999). The health of grandparents raising grandchildren: results of a national study. Am J Public Health.

[36] Musil CM, Gordon NL, Warner CB, Zauszniewski JA, Standing T, Wykle M (2011). Grandmothers and caregiving to grandchildren: continuity, change, and outcomes over 24 months. Gerontologist.

[37] Choi L. Financial stress and its physical effects on individuals and communities. Community Dev Invest Rev [Internet]. 2009;120–2. http://www.frbsf.org/community-development/files/choi.pdf

[38] Lee C-YS, Financial Stress GJA (2011). Parental depressive symptoms, parenting practices, and children’s externalizing problem behaviors. Underlying Processes.

[39] Guan N, Guariglia A, Moore P, Xu F, Al-Janabi H. Financial stress and depression in adults: A systematic review. PLoS One [Internet]. 2022;17(2 Febuary):1–20. 10.1371/journal.pone.0264041 10.1371/journal.pone.0264041 PMC8863240 35192652

