# Supplementary material for: Through the eyes of grandparents: an in-depth exploration of the nexus between grandchild caring and the psychological well-being of older grandparents
Source: BMC Geriatr. 2024 May 5;24:399. doi: 10.1186/s12877-024-04998-z (PMC11070074; doi:10.1186/s12877-024-04998-z)
Supplement: Supplementary file 1 — Supplementary Material 1 [file 12877_2024_4998_MOESM1_ESM.docx]

# Appendix 1: Word from word clouds

| All Households | | | MGH | | | SGH | | |
| --- | --- | --- | --- | --- | --- | --- | --- | --- |
| Word | % | Similar Words | Word | % | Similar Words | Word | % | Similar Words |
| happy | 3.4 | happiness, happy | happy | 4.9 | happiness, happy | tension | 3.4 | tension, tensions |
| tension | 3.0 | tension, tensions | tension | 2.7 | tension | think | 3.4 | think, thinking, thinks |
| think | 3.0 | think, thinking, thinks | think | 2.2 | think, thinking | worried | 1.9 | worried, worries, worry, worrying |
| worry | 2.1 | worried, worries, worry, worrying | worry | 2.2 | worries, worry | happy | 1.9 | Happiness, worry |
| children | 1.3 | children | children | 1.8 | children | depression | 1.5 | depressed, depression |
| depressed | 1.3 | depressed, depression | much | 1.8 | much | sleep | 1.5 | sleep |
| peace | 1.3 | peace | poor | 1.8 | poor | time | 1.5 | time |
| poor | 1.3 | poor | pressure | 1.8 | pressure | well | 1.5 | well |
| anyone | 1.1 | anyone | today | 1.8 | today | discomfort | 1.1 | discomfort |
| grandson | 1.1 | grandson, grandsons | anyone | 1.3 | anyone | grandsons | 1.1 | grandson, grandsons |
| like | 1.1 | like | blessed | 1.3 | blessed | headaches | 1.1 | headaches |
| things | 1.1 | thing, things | child | 1.3 | child | night | 1.1 | night, nights |
| well | 1.1 | well | conversation | 1.3 | conversation | peace | 1.1 | peace |
| child | 0.8 | child | friend | 1.3 | friend | responsibility | 1.1 | responsibilities, responsibility |
| empty | 0.8 | empty | granddaughter | 1.3 | granddaughter | anyone | 0.8 | anyone |
| however | 0.8 | however | however | 1.3 | however | burden | 0.8 | burden |
| much | 0.8 | much | like | 1.3 | like | challenges | 0.8 | challenges, challenging |
| never | 0.8 | never | mother | 1.3 | mother | children | 0.8 | children |
| pressure | 0.8 | pressure | never | 1.3 | never | course | 0.8 | course |
| sleep | 0.8 | sleep | partner | 1.3 | partner | daily | 0.8 | daily |

**Interview guidelines and checklist:**

| IDENTIFICATION | | | | | | | | |
| --- | --- | --- | --- | --- | --- | --- | --- | --- |
|  |  |  |  |  |  |  |  |  |
| LOCATION OF THE OLDER GRANDPARENT_________________________________________ | | | | | | | | |
| Address of the Respondent responsible for grandchild caring____________________________ | | | | | | | | |
| _______________________________________________________________________________ | | | | | | | | |
| SERIAL NUMBER |  |  |  |  |  |  |  |  |
|  |  |  |  |  |  |  |  |  |
| Contact no. of Researcher: Guide: Respondent: | | | | | | | | |
|  |  |  |  |  |  |  |  |  |
|  |  |  |  |  |  |  |  |  |
| INTERVIEW DATE | Date | | Month | | Year | | | |
|  |  |  |  |  |  |  |  |  |
|  |  |  |  |  |  |  |  |  |
| RESULT STATUS OF THE INDIVIDUAL QUESTIONNAIRE | | | | | | | | |
| TOTAL PERSON IN HOUSEHOLD ….............................................. | | | | | | |  |  |
| TOTAL MALE….......................................................................... | | | | | |  |  |  |
| TOTAL FEMALE …...................................................................... | | | | | |  |  |  |
|  |  |  |  |  |  |  |  |  |
| INVESTIGATOR…..................................... | | | | FIELD EDITED BY…............................................. | | | | |

**Informed consent**

I would first like to thank you for taking time and ensuring for meeting and talking with me today. I am Papai Barman, a student of International Institute for Population Science (IIPS) which is located at Govandi Station Road, Deonar, Mumbai, in Maharashtra 400088 and I would like to talk to you about views and experiences on caring and caring responsibility for your grandchild. The purpose of the study is to understand your perspectives on process, reasons, motivation and challenges you have faced for caring to your grandchild. Your views and experiences will be used for academic research purpose only. Your participation will be very useful. The interview will be for around one hour and I will be taking notes and recording your responses because I don’t want to miss of your any comments during interview. Since I will use the tape machine, you will try to speak a little louder so that it will be easier for me to record. All responses that will be records and noted will be kept in confidential which means your given responses, your name and experiences, will be used for research purpose only and will not be shared to other members or organization. I also ensure you that all information that will be included in research will not identify you as a respondent. I let you know about the right during interview that whenever you want to withdraw from interview, you are free to do this at any time of during interview. Further, if you do not want to answer for a question or several questions, you may skip the question(s). You also have right to seek clarification and information about any aspect of the research. I also let you know that there is no any benefits and risks for your participation in this interview.

Do you have any question or doubt about that I have explained to you now?

Are you willing to participate in this interview?

I would like to thank you again for willing to participate and share the information.

SIGNATURE OF THE INTERVIEWR_____________________ DATE____________

1. CONSENT WITH SIGNATURE…………. TO BE INTERVIEWD

2. CONSENT WITHOUT SIGNATURE……. TO BE INTERVIEWD WITHOUT SIGNATUR

3. REFUSAL…………………………………. END

**Identification Information**

- 1. Name ______________________________
  2. Age
  3. Sex: Male Female
  4. Ward No. / House No. & Address

____________________________________________________________________

____________________________________________________________________

- 1. Name of the Area
  2. City/Town/Village/Sub-Urban ______________________
  3. Pin code ________________________
  4. District _________________________

1.9 State ___________________________

**Socio-Economic and Demographic Background**

- 1. Religion ________________________
  2. Caste ________________________
  3. Language ______________________
  4. Current Marital Status ________________________
  5. Highest level of education _______________________
  6. In-migrated from _____________________________
  7. Working Status _________________________
  8. Occupation:
     1. Principle Occupation _______________
     2. Any other ________________________
  9. Number of the family members ____________________

2.10 Number of adult child(ren) _______________________

3.1 Number of Grandchild __________________

3.2 Number of Grandchild for whom you are responsible for caring____________

3.3 Age _______________________

3.4 Sex _______________________

1: How have you taken decision for grandchild parenting?

2: What are the motivating factors for grandchild parenting?

3. How do you manage all types of household expenditure?

4. How do you feel about caring, sometimes it is a lot of responsibility and at the same time it is a pleasure to spend time and get social, physical, and mental support with grandchild? What is in your case?
